# Relationship between maintenance of occlusal support achieved by home‐visit dental treatment and prognosis in home‐care patients—a preliminary study

**DOI:** 10.1111/ggi.14482

**Published:** 2022-09-20

**Authors:** Takeshi Kikutani, Noriaki Takahashi, Takashi Tohara, Hiroyasu Furuya, Kumi Tanaka, Kimiko Hobo, Tomoko Isoda, Tomoko Fukui

**Affiliations:** ^1^ The Nippon Dental University, Tama Oral Rehabilitation Clinic Tokyo Japan; ^2^ Division of Rehabilitation for Speech and Swallowing Disorders The Nippon Dental University Tokyo Japan; ^3^ Suginami Oral Health and Care Center Tokyo Japan

**Keywords:** denture, home‐visit dental treatment, occlusal support, older adults, prognosis

## Abstract

**Aim:**

To determine whether occlusal maintenance and reconstruction by dental intervention is associated with the prognosis of older home‐care patients.

**Methods:**

The study participants were 289 older home‐care patients (101 males, mean age 82.2 ± 7.7 years) who received visiting dental treatment from dental clinics in the region between 2012 and 2018. The participants were followed up for 1000 days after receiving the necessary dental treatment in a home‐visit setting. The participants were divided into three groups: those with natural tooth occlusion, those whose molar occlusion was maintained or reconstructed by dentures, and those whose occlusion was not reconstructed. Factors associated with prognosis were determined using the Cox proportional hazard model, with occlusal status, comorbidities, the activity of daily living, and residence status as explanatory variables.

**Results:**

In the overall population, occlusal status (hazard ratio [HR] of those with occlusal disintegration versus those with natural tooth occlusion: 2.1, confidence interval [95% CI]: 1.18–3.82) and age (HR: 2.28, 95% CI: 1.44–3.61) were identified as significant factors.

In the group of participants aged <85 years, only occlusal status (HR of those with occlusal disintegration versus those with natural tooth occlusion: 3.4, 95% CI: 1.34–8.68) was a significant factor. In the group of participants aged ≥85 years, occlusal status was not significantly associated with prognosis.

**Conclusions:**

The maintenance and acquisition of occlusal support achieved by dental treatment contribute to improved prognosis in older patients younger than 85 years requiring home nursing care. **Geriatr Gerontol Int 2022; 22: 976–981**.

## Introduction

The maintenance of oral function is essential for older patients to maintain a good prognosis and quality of life. Maintenance of the remaining number of teeth leads to the maintenance of occlusal support and healthy oral function.^1^ Extensive studies have suggested an association between tooth loss and prognosis.^2^ A systematic review involving community‐dwelling older individuals showed a significant association between tooth loss and the incidence of all‐cause death.^3^, ^4^ Lee *et al*. reported that tooth loss was associated with all‐cause mortality, in addition to the incidence of myocardial infarction, heart failure, and stroke, in a quantity‐dependent manner.^5^ In follow‐up studies of community‐dwelling older individuals conducted by Nomura *et al*.^6^ and Hiratsuka *et al*.,^7^ the number of remaining teeth, as well as nutritional indicators such as serum albumin levels, were identified as prognostic factors. These findings suggest an association between tooth loss and prognosis in older individuals, indicating that regular oral management is effective in the prevention, early detection, and treatment of dental diseases. A few reports have shown that the maintenance of continuous occlusal support with dentures has been associated with prognosis;^8^ however, none of the related studies examined the effect of denture prosthetic treatment on the reconstruction or maintenance of occlusal support with dentures.

Continuous dental treatment has been shown to be effective in maintaining physical function in community‐dwelling older individuals.^9^ On the other hand, age‐related decline in physical and cognitive functions makes it difficult for these individuals to receive outpatient dental treatment, necessitating visiting dental treatment.^10^ However, medical services that can be provided through home‐visit care are limited compared with those available through outpatient care, because there is a limit to the amount of medical equipment that can be brought in owing to the difficulty of securing a sufficiently hygienic environment. However, visiting care is superior to outpatient care in many aspects; for example, it allows dentists to understand the patient's functional capacity and to provide lifestyle guidance to family members and caregivers.^11^ For these reasons, providing dental treatment at patients' homes is considered to be effective for the oral management of community‐dwelling older individuals. Nevertheless, the effectiveness of visiting dental treatment has not been adequately investigated.^12^

The objective of this study was to investigate the association of the acquisition or maintenance of occlusal support achieved by dental intervention with prognosis in older home‐care patients and to determine whether occlusal maintenance or reconstruction achieved by dental intervention contributes to improved prognosis in these patients.

## Methods

### Participants

**Figure. 1:**
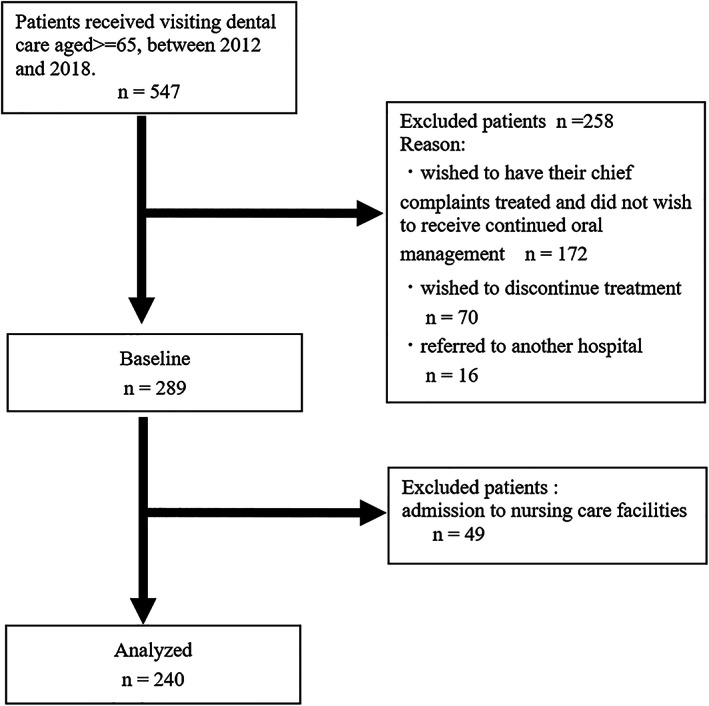
Flow chart of participant selection.

Figure 1 is a flow chart of participant selection. This study included older individuals aged ≥65 years who received visiting dental treatment from community dental clinics between 2012 and 2018. A total of 547 patients (201 males, mean age 82.4 ± 7.6 [65–101] years; and 346 females, mean age 86.3 ± 7.9 [65–108] years) received visiting dental treatment during this period. Of these, 172 patients who only wished to have their chief complaints treated and did not wish to receive continued oral management (62 males, mean age 84.0 ± 7.3 [65–96] years; and 110 females, mean age 86.6 ± 8.1 [65–108] years), 70 patients who wished to discontinue treatment (27 males, mean age 83.1 ± 8.2 [65–101] years; and 43 females, mean age 86.3 ± 7.6 [65–102] years), and 16 patients who were referred to another hospital (11 males, mean age 80.3 ± 8.3 [67–93] years; and 5 females, mean age 81.0 ± 13.6 [66–94] years) were excluded, and the remaining 289 patients (101 males, mean age 82.2 ± 7.7 [66–99] years; and 188 females, mean age 85.8 ± 7.7 [65–101] years) were included in the follow‐up study.

### Intervention

Data on the status of occlusal support after the necessary dental treatment were used to evaluate occlusal support. For participants who had lost occlusal support before the intervention, dentures were fabricated to restore occlusion. In participants requiring tooth extraction owing to severe periodontal disease, severe caries, or other problems, tooth extraction was followed by the fabrication of dentures with the aim of restoring occlusal support. For those who used dentures that were incompatible, denture adjustment was performed or new dentures were made so that these individuals could continuously use dentures. Subsequently, visiting dental treatment was continuously provided at monthly intervals.

### Baseline parameters

The first day of follow‐up was the day of the first visit for participants with natural tooth occlusion, and at 3 months after the completion of denture fabrication for those who did not have natural tooth occlusion and for those for whom the fabrication of a new denture was considered necessary owing to denture incompatibility.

Sex and age at the start of follow‐up were recorded. Activities of daily living (ADL) were evaluated using the Japanese Bedriddenness Rank (BR).^13^ BR is an ADL assessment tool developed for the assessment of eligibility for coverage by the Japanese health insurance and nursing care insurance systems and is significantly correlated with the Barthel Index and Katz Index.^13^, ^14^

### Comorbidities

Comorbidities were evaluated according to the Charlson Risk Index, with a score of 0 indicating low risk, a score of 1 or 2 indicating medium risk, a score of 3 or 4 indicating high risk, and a score of 5 indicating very high risk.

### Diagnosis of occlusal support

Occlusal support zones (i.e., occlusal stability) in the left and right premolar/molar regions were evaluated using the Eichner classification.^15^ In addition to occlusal support with natural teeth, occlusal support with fixed, removable, and implant dentures was also evaluated. Participants classified as Eichner group A (having occlusive support in all four zones), group B1 (having three supporting zones), or group B2 (having two supporting zones) were considered to have occlusal support with natural teeth, while those classified as Eichner group B3 (having one supporting zone) or group C (having no occlusive support) were considered to have no occlusal support with natural teeth. For those without occlusal support, dentures were fabricated, as these were considered necessary to restore occlusion. Three months after denture fabrication, the occlusal relationship with natural teeth and functional teeth including dentures was evaluated in the same manner as described above, taking into account the status of denture use. Based on this evaluation, participants were classified into three groups: those with natural tooth occlusion (i.e., natural tooth occlusion group); those who had no molar occlusion but achieved the restoration of occlusion with dentures or those who continued to use dentures with adjustment (i.e., denture maintenance and restoration group); and those for whom dentures were fabricated owing to lack of occlusion, but could not use them and did not have restored occlusion (i.e., no occlusal support group).

### Prognosis

Participants who were hospitalized or died during the follow‐up period were considered to have a poor prognosis, except for those who could not be followed because of withdrawal owing to deconditioning or admission to nursing care facilities. Participants who continued to receive dental treatment beyond the follow‐up period were considered to have a good prognosis.

### Sample size calculation

The sample size was calculated using G*Power 3.1.9.2 Statistical Power Analyses (University of Dusseldorf, Germany), with a type I error of 0.05, a type II error of 0.2, and an effect size of the medium. Assuming that 30% of patients would stop receiving dental treatment owing to hospitalization or death during 1 year (based on unpublished data), it was estimated that at least 217 patients would be required.

### Analysis methods

The participants were followed up for up to 1000 days. Associations between the maintenance/restoration of occlusal support with natural teeth or dentures, each factor, and prognosis were examined using the chi‐square test and Cox proportional hazard model. Owing to an anticipated age effect, participants were divided into two age groups (<85 and ≥ 85 years) for comparison. All statistical analyses were performed using the IBM SPSS Ver. 23 for Windows.

## Results

A total of 49 participants (19 males and 30 females; mean age 84.3 ± 7.2 years) were lost to follow‐up owing to admission to nursing care facilities during the follow‐up period, leaving 240 participants (82 males and 158 females) included in the final analysis (Figure 1).There were 60 participants (22 males and 38 females, mean age 85.6 ± 6.9 years) who completed treatment on an inpatient basis, and 36 participants (12 males and 24 females, mean age 88.8 ± 6.8 years) who died during the follow‐up period. Thus, these 96 participants (34 males and 62 females, mean age 86.8 ± 7.0 years) who were hospitalized or died were considered to have a poor prognosis. The baseline characteristics of the participants are shown in Table 1. A total of 144 participants (48 males and 96 females, mean age 83.1 ± 8.3 years) continued treatment beyond the follow‐up period, and thus were considered to have a good prognosis. The median duration of survival was 300 days (interquartile range [IQR]: 123–582).

**Table 1:** Characteristics and comparison of outcome

		Overall *n* = 240	Good prognosis *n* = 144	Poor prognosis *n* = 96	*p*‐value
	Age (years)	84.6 ± 8.0	83.1 ± 8.3	86.8 ± 7.2	< 0.001
	<85 years	115(47.9)	84(58.3)	31(32.3)	< 0.001
	≥85 years	125(52.1)	60(42.7)	65(67.7)	
Sex	Male	82(34.2)	48(33.3)	34(35.4)	0.739
ADLs	J	10(4.2)	9(6.3)	1(1.0)	0.249
	A	134(55.8)	77(53.5)	57(59.4)	
	B	61(25.4)	37(25.7)	24(25.0)	
	C	35(14.6)	21(14.6)	14(14.6)	
Charlson Comorbidity Index	Low	87(34.4)	54(37.5)	33(34.4)	0.151
	Medium	116(48.3)	63(43.8)	53(55.2)	
	High	34(14.2)	24(16.7)	10(10.4)	
	Very high	3(1.3)	3(2.1)	0(0.0)	
Residential status	Alone	49(20.4)	27(18.9)	22(22.9)	0.433
Occlusal support	OSNT	116(48.3)	82(57.0)	34(35.4)	0.003
	OSFT	94(39.2)	49(34.0)	45(46.9)	
	NO	30(12.5)	13(9.0)	17(17.7)	

Abbreviations: ADLs, Japanese classifications of activities of daily living; A, house‐bound; B, chair‐bound; C, bed‐bound; J, normal; NO, no occlusal; OSNT, occlusal support with natural teeth; OSFT, occlusal support with functional teeth.

Table 2 shows the relationships between occlusal support and each item. The median age was 85 years (IQR: 70–91). At the start of follow‐up, 116 participants had occlusal support with natural teeth, and 124 did not. At the same time point, 30 participants had lost their occlusal support owing to their inability to use dentures or difficulty in wearing dentures. We then analyzed the relationships between occlusal status and each factor. Significant associations were found for age group (*p* = 0.001) and residential status (*p* = 0.024).

**Table 2:** Relationships between occlusal support and each item

		OSNT *n* = 116	OSFT *n* = 94	NO *n* = 30	*p*‐value
Age (years)	<85 years	68(58.6)	31(33)	16(53.3)	0.001
	≥85 years	48(41.4)	63(67)	14(46.7)	
Sex	male	35(30.2)	39(41.5)	8(26.7)	0.149
ADLs	J	4(3.4)	5(5.3)	1(3.3)	0.250
	A	60(51.7)	59(62.8)	15(50)	
	B	31(26.7)	23(24.5)	7(23.3)	
	C	21(18.1)	7(7.4)	7(23.3)	
Charlson Comorbidity Index	Low	49(42.2)	31(33)	7(23.3)	0.499
	Medium	50(43.1)	48(51.1)	18(60)	
	High	15(12.9)	14(14.9)	5(16.7)	
	Very high	2(1.7)	1(1.1)	0(0.0)	
Residential status	alone	17(14.7)	21(22.3)	11(36.7)	0.024

Abbreviations: ADLs, Japanese classifications of activities of daily living; NO, no occlusal; OSNT, occlusal support with natural teeth; OSFT, occlusal support with functional teeth.

**Figure. 2:**
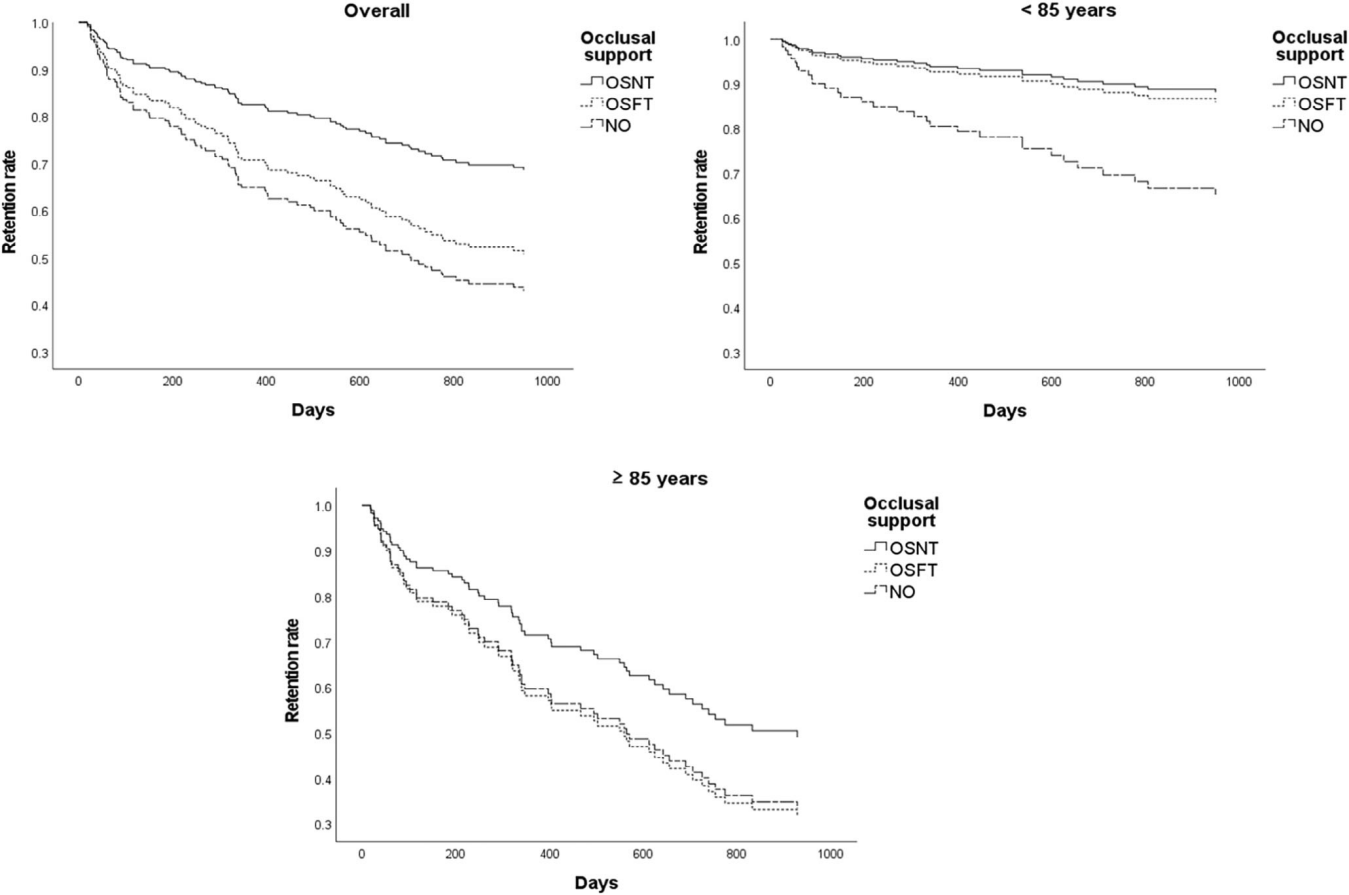
Retention rate of visiting dental treatment according to occlusal support. NO, no occlusal; OSNT, occlusal support with natural teeth; OSFT, occlusal support with functional teeth.

Table 3 and Figure 2 show the Cox proportional hazard model analysis. In the overall population, a Cox proportional hazard model analysis showed a significant association for occlusal status (*p* = 0.033), where the hazard ratio (HR) of those with occlusal disintegration versus those with natural tooth occlusion was 2.12 (confidence interval [95% CI]: 1.18–3.82). A significant association was also found for age group, with an HR of 2.28 (95% CI: 1.44–3.61, *p* < 0.001).

**Table 3:** Cox proportional hazard model analysis

		Overall *n* = 240		<85 years *n* = 115		≥85 years *n* = 125	
		Unadjusted hazard ratio (95% CI)	*p*‐value	Adjusted hazard ratio (95% CI)	*p*‐value	Adjusted hazard ratio (95% CI)	*p*‐value
	Age (years)	2.28(1.44–3.61)	<0.001	‐		‐	
ADLs	J	1.00(Reference)		1.00(Reference)		1.00(Reference)	
	A	4.43(0.60–32.95)	0.146	31 018.22(0–3.64)	0.911	1.25(0.16–9.67)	0.833
	B	5.18(0.66–40.50)	0.117	41 570.34(0–4.89)	0.909	1.36(0.16–11.52)	0.780
	C	5.23(0.66–41.68)	0.119	39 969.59(0–4.70)	0.909	1.46(0.16–13.08)	0.733
Charlson Comorbidity Index	Low	1.00(Reference)		1.00(Reference)		1.00(Reference)	
	Medium	1.36(0.87–2.11)	0.174	2.39(1.02–5.57)	0.044	1.14(0.67–1.95)	0.623
	High	0.66(0.31–1.37)	0.262	1.09(0.31–3.85)	0.890	0.53(0.21–1.36)	0.188
	Very high	0.00(0.00–1.73)	0.964	0.00(0–2.37)	0.963	‐	
Residential status	Alone	2.26(1.44–3.61)	0.242	1.18(0.46–3.03)	0.726	1.34(0.71–2.53)	0.370
Occlusal support	OSNT	1.00(Reference)		1.00(Reference)		1.00(Reference)	
	OSFT	1.51(0.95–2.41)	0.080	1.20(0.48–2.98)	0.696	1.61(0.91–2.85)	0.100
	NO	2.12(1.18–3.82)	0.010	3.41(1.34–8.68)	0.010	1.54(0.67–3.55)	0.311

Abbreviations: ADLs, Japanese classifications of activities of daily living; NO, no occlusal; OSNT, occlusal support with natural teeth; OSFT, occlusal support with functional teeth.

Among those aged <85 years, 31 participants had poor prognosis. A Cox proportional hazard model analysis showed a significant association for occlusal status (*p* = 0.032), with the HR of those with occlusal disintegration versus those with natural tooth occlusion being 3.41 (95% CI: 1.34–8.68, *p* = 0.010). In contrast, no significant effect of occlusal status was observed in the denture maintenance and restoration group (HR: 1.20, 95% CI: 0.48–2.98, *p* = 0.696).

Among those aged ≥85 years, 65 had poor prognosis. A Cox proportional hazard model analysis showed no significant association between the occlusal status and prognosis.

## Discussion

This study is the first to demonstrate that the maintenance and reconstruction of occlusal support achieved by dental intervention are associated with prognosis in older patients requiring home nursing care. The results of this study revealed that the maintenance of occlusal support affects the prognosis of older patients in need of nursing care. Especially in patients younger than 85 years, the maintenance and restoration of occlusal support with dentures achieved by prosthodontic treatment had a positive effect on prognosis.

Denture fabrication is performed to restore occlusal support in patients with collapsed occlusal support. If the area of occlusal support including dentures is associated with the prognosis of older home‐care patients, it will be possible to emphasize the effectiveness of prosthodontic treatment and regular oral management provided in a home‐visit setting. Only a few studies have evaluated and demonstrated the association between the reconstruction of occlusal support with dentures and survival prognosis. Among studies involving community‐dwelling older adults, a follow‐up study of randomly selected community‐dwelling older individuals conducted by Yoshida *et al*. showed a significant difference in survival prognosis between those without occlusal support who did not use dentures and those who did use dentures.^16^ Maekawa *et al*. examined the effect on survival prognosis of not only the number of remaining teeth, but also the number of functional teeth, including prostheses and implants, and found that the survival prognosis improved with an increasing number of teeth.^17^ However, all of these studies are prospective or retrospective cohort studies without intervention in community‐dwelling older people. The present study is important in that it involved patients who underwent the intervention.

The results of the present study demonstrated that the presence of occlusal support and the maintenance/acquisition of occlusion achieved by visiting dental treatment had a positive effect on prognosis. The maintenance of occlusal support is known to be important for the preservation of swallowing function.^18^ Furthermore, the maintenance of occlusal support by natural teeth or dentures may contribute to prognosis by preventing aspiration and nutritional deficiencies. It is well known that the significant associations between occlusal support and nutritional status, and dietary variation.^19^, ^20^, ^21^ Furthermore, Kanehisa *et al*. reported that prosthodontic treatments can improve nutritional status in institutionalized older people.^22^ It is also known that the presence of occlusal support affects posture stabilization,^23^ and is important for ADL maintenance in older individuals in need of nursing care.^24^ These facts may also explain the effect of occlusal maintenance/restoration on prognosis.

The present study showed differences in some results between participants aged <85 years and those aged ≥85 years. Suzuki *et al*. demonstrated that among older patients requiring nursing care, the presence of occlusal support was associated with prognosis in those with relatively preserved ADL, while nutritional status was associated with prognosis in those with impaired ADL.^25^ Fujiwara described how the relative value of the presence of teeth varies depending on the patient's condition.^26^ The results of the present study suggest that the maintenance of occlusal support and its acquisition achieved by prosthodontic treatment improve patient prognosis, but that this benefit would be lost with the increasing age of the patients.

This study has several limitations. First, it remains unclear how occlusal support and the acquisition of occlusion achieved by denture use affected prognosis. A significant association has been demonstrated between denture use and independence in oral hygiene behavior.^27^ In addition, Taji *et al*. found that cognitive decline affected patients' ability to use dentures.^28^ Thus, these factors that make denture use difficult are consistent with the factors associated with poor survival prognosis. These findings suggest that the ability to use dentures, which is equivalent to the ability to re‐acquire occlusion through denture prosthetic treatment, might be a potential predictor of mortality. Second, we did not analyze differences in the denture maintenance and restoration group between participants who had their existing dentures repaired or relined and those who had new dentures made. If these were analyzed, the results might have differed from the present results. However, the restoration and maintenance processes were numerous and difficult to subgroup and analyze. Third, among the participants with poor prognosis, those who were hospitalized could not be followed. For participants who were admitted to hospital but were discharged to home in a short period, visiting dental treatment and follow‐up were resumed, whereas those hospitalized for an extended period could not be followed. Because the patients included in this study were older at the start of follow‐up, those who were hospitalized for a long period were considered to have a poor prognosis. Fourth, the participants of this study were patients treated at a single medical institution. Although it is an advantage of this single‐center study that the quality and methods of dental interventions could be standardized, further investigations are needed in a larger‐scale, multi‐center setting.

## Conclusion

The maintenance and acquisition of occlusal support achieved by dental treatment are associated with prognosis in older patients requiring home nursing care. Dental treatment provided in a home‐visit setting contributes to improved prognosis in older patients younger than 85 years requiring nursing care.

## Author contributions

Takeshi Kikutani, Noriaki Takahashi, Takashi Tohara, Hiroyasu Furuya, Kumi Tanaka, Kimiko Hobo, Tomoko Isoda and Tomoko Fukui were involved in study design and data interpretation. Takeshi Kikutani and Kumi Tanaka were involved in the data analysis. All authors critically revised the report, commented on drafts of the manuscript, and approved the final report.

## Conflict of interest

The authors have no conflicts of interest to declare.

## Ethics statement

The data are not publicly available due to privacy or ethical restrictions.

## Data Availability

This study was conducted after obtaining approval from the Nippon Dental University School of Life Dentistry Ethics Committee (approval code: NDU‐T2021‐20).
